# Physiological changes for drought resistance in different species of *Phyllanthus*

**DOI:** 10.1038/s41598-018-33496-7

**Published:** 2018-10-11

**Authors:** Elenilson G. Alves Filho, Luiza N. Braga, Lorena Mara A. Silva, Fábio R. Miranda, Ebenézer O. Silva, Kirley M. Canuto, Maria Raquel Miranda, Edy S. de Brito, Guilherme J. Zocolo

**Affiliations:** 1 Embrapa Agroindustria Tropical, Fortaleza, CE Brazil; 2 Departamento de Agronomia, Universidade Federal do Ceará, Fortaleza, CE Brazil; 3 Departamento de Bioquímica e Biologia Molecular, Universidade Federal do Ceará, Fortaleza, CE Brazil

## Abstract

The *Phyllanthus* genus is widely distributed in tropical and subtropical areas of the world and present several pharmacological applications. Drought is a restrictive factor for crop development and production, and is becoming a severe problem in many regions of the world. The species *Phyllanthus amarus* and *Phyllanthus niruri* were subjected to drought stress for varying periods of time (0, 3, 5, 7, and 10 days), and afterwards, leaves were collected and evaluated for physiological and biochemical responses, such as oxidative stress markers and drought-associated defense mechanisms. Results show that *P. amarus* has an endogenously higher level of variables of the oxidative/antioxidant metabolism, and *P. niruri* presents the most significant changes in those variables when compared to control and stressed plants. For both *Phyllanthus* species, drought stress induces higher levels of organic acids such as malic, succinic, and citric acids, and amino acids such as proline, GABA, alanine, and valine. Moreover, *P. niruri* plants respond with greater glucose and corilagin contents. Therefore, considering the evaluated metabolic changes, *P. amarus* is better adapted to drought-stress, while *P. niruri* presents an acclimation strategy that increases the corilagin levels induced by short-term drought stress.

## Introduction

Membrane transport of plants under drought stress is disturbed, with alterations in the nucleic acid structure and activity. As a result, damages to cell structures including nucleic acid and proteins are observed, and consequently, alterations in genetic programming occur^1,2^, which trigger oxidative processes. Therefore, homoeostasis is pursued through changes in metabolism, which decreases or increases the content of primary and secondary metabolites in cells. Proline, for instance, is an amino acid associated with tolerance to drought stress, conferring osmotic adjustment and protection against protein denaturation^3^. Recently, another study reported that short-chain organic acids accumulate in response to drought stress^4^. An increase in non-protein amino acid such as GABA (gamma-aminobutyric acid) has been observed in different plant tissues in response to drought stress^5^. In addition, in 2014, Król and coworkers reported that drought stress inhibits the total synthesis of phenolic compounds in grapevine leaves^6^. However, the responses to abiotic stresses such as drought vary with plant species, developmental stage, duration, and severity^7,8^. However, low levels of stress may induce positive changes such as physiological stimulation of beneficial responses. Therefore, controlled drought stress could be used as a tool to enhance the phytochemical levels of either a whole plant or its organs^9^.

The *Phyllanthus* genus is widely distributed in tropical and subtropical areas all around the world. In Brazil, 88 species of *Phyllanthus* are found^10^. It is used for medicinal purposes in several countries, such as for the treatment of hepatitis B, hypertension, dropsy, sore throat, jaundice, renal calculus, kidney and gallbladder stones, malaria, liver cancer and viral infections^11–13^. *P. niruri* is commonly found in southeast and central Brazil^14^ in damp and shaded locations^15^, while *P. amarus* is commonly found in northeastern Brazil, especially Ceara state^16^, and exhibits great adaptability to sandy or sandy loam soils^15^. In some works, *P. amarus* and *P. niruri* are claimed to be the same species. However, in 2013, Sprenger and Cass used liquid chromatography coupled with ion trap tandem mass spectrometry (LC–IT-MS^n^)^17^ to show distinct chemical signatures for *P. amarus*, *P. stipulatus*, *P. niruri*, and *P. tenellus*. In our previous research^18^, we found a higher content of total phenolic compounds in *P. niruri* than in *P. amarus* extracts, supported with UPLC–QTOF–MS^E^, that also shows a different chemical signature for both species. In addition, according to the Brazilian Pharmacopeia^19^, the indicated species to use for pharmacological purposes correspond to *Phyllanthus niruri* and *Phyllanthus tenellus*.

Non-targeted metabolite profiling using ^1^H *q*NMR spectroscopy and UPLC-HRMS generates unique fingerprints of the samples^20^. These techniques are often combined with chemometric tools, allowing a comprehensive view on the metabolome and making possible the understanding of the physiological state of an organism. The aim of the present study was to investigate the physiological and oxidative responses of *Phyllanthus amarus* and *Phyllanthus niruri* when subjected to drought stress.

## Results

### Drought stress effects on oxidative/antioxidant metabolism

**Figure 1:**
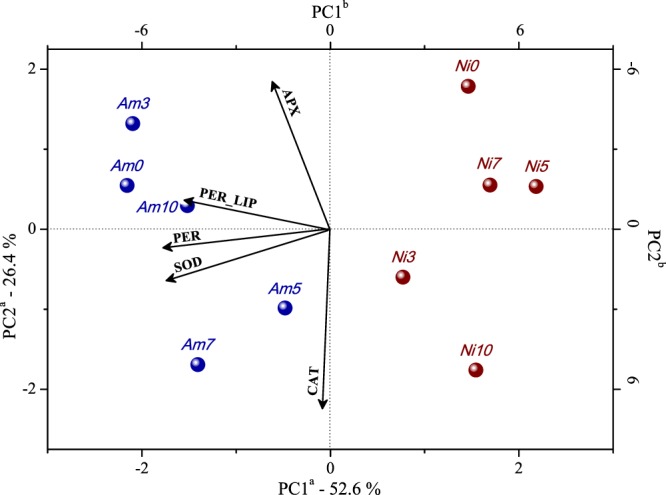
PCA biplot of physiological responses of *P. amarus* (blue) and *P. niruri* (red) under different drought stress. ^a^Axes refer to scores from the samples with explained variance on each Principal Component; ^b^axes refer to loadings; selected variables are represented as vectors from the origin. Scores legend: number 0 corresponds to the non-stressed plant, 3 corresponds to three days of hydric stress, 5 corresponds to five days of hydric stress, 7 corresponds to seven days of hydric stress, and 10 corresponds to ten days of hydric stress. Loadings legend: APX - ascorbate peroxidase; PER - hydrogen peroxide; PER_LIP - lipid peroxidation degree; SOD - superoxide dismutase; and CAT - catalase.

Physiological responses of *Phyllanthus* species (*P. amarus* and *P. niruri*) submitted to drought stress were evaluated through principal component analysis (PCA), and the results are illustrated in Fig. 1. At negative scores of PC1 (which accounts for 52.6% of the total variance), *P. niruri* presents lower levels of PER, PER_LIP, SOD, and APX activity than *P. amarus* (at positive scores of PC1). In addition, the CAT activity does not vary significantly between the species, but increases in stress-treated *P. amarus* after five and seven days (*Am5 and Am7*, respectively) and *P. niruri* after three and ten days (*Ni3* and *Ni10*, respectively). *P. niruri* presents the most significant changes in oxidative/antioxidant activities when comparing control (*Ni0*) and the stress-treated plants (*Ni3*, *Ni5*, *Ni7*, and *Ni10*) in response to drought. This result shows that *P. niruri* exhibits the most dramatic metabolic changes while trying to adjust to changes in the water status of the environment, under the imposed conditions.

### Chemometric evaluation of the NMR data

**Figure 2:**
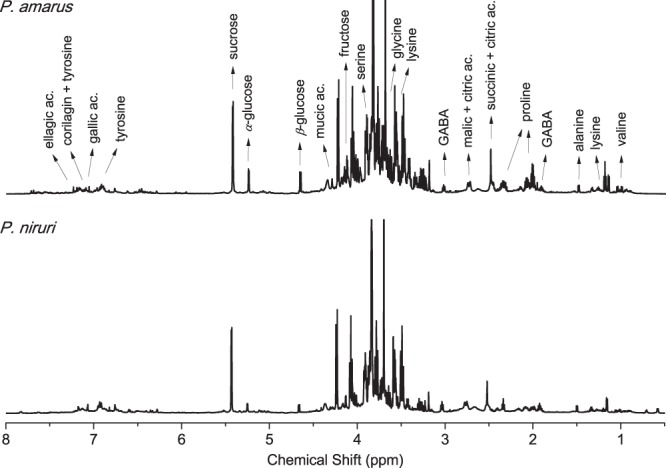
^1^H NMR spectra of leaves from *P. amarus* (top) and *P. niruri* (bottom).

For an overall metabolic comprehension of *P. amarus* and *P. niruri*, a comparison between the ^1^H NMR spectra from non-hydric stressed plants is presented in Fig. 2. In general, the ^1^H NMR spectra presents compounds in three different regions: aliphatic alicyclic, allylic, *β*-substituted aliphatic, and alkyne protons (*δ* 0.5–3.1); carbinolic, olefinic, *α*-monosubstituted, and *α*-disubstituted aliphatic protons (*δ* 3.1–5.5); and alkene, aromatic, heteroaromatic, and aldehydic protons (*δ* 6.2–9.2), which shows that the *Phyllanthus* leaves comprises a high level of aliphatic and aromatic structures (for more information, see the Support Information). Results from UPLC-HRMS analysis were complementary to the characterization of certain compounds, as ellagic acid and corilagin.

**Figure 3:**
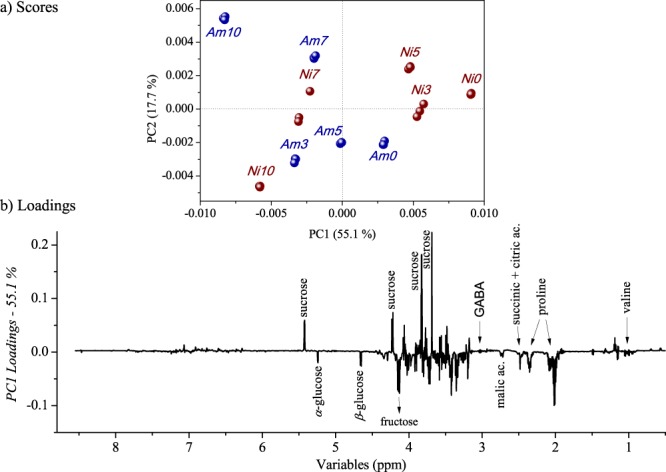
(**a**) PC1 × PC2 scores plot for *P. amarus* and *P. niruri* submitted to different hydric stress conditions, and (**b**) PC1 loadings plotted in line form. Legend: Number 0 corresponds to the non-stressed plant, 3 corresponds to three days of hydric stress, 5 corresponds to five days of hydric stress, 7 corresponds to seven days of hydric stress, and 10 corresponds to ten days of hydric stress.

Due to the high complexity of the dataset, chemometric analysis was applied to investigate differences in the leaf composition of both species of *Phyllanthus* caused by drought conditions. Initially, hierarchical cluster analysis (HCA) was applied, which presented natural clusters into two-dimensional space (dendrogram available as Fig. 1 in Supplementary Information). Three important clusters are observed at a similarity index of 0.552: one group comprises most stressed plants (regardless of the *Phyllanthus* species); the second group includes intermediately-stressed *P. amarus* leaves; and the third group comprises *P. niruri* within lower or non-stressed leaves. On the other hand, with a similarity index of zero (no similarity), a group comprising *P. amarus* and *P. niruri* faced the greatest hydric stress. The HCA evaluation was important to comprehend the separation tendencies observed in PCA, which was applied to assist in the interpretation of the multivariate data. Figure 3a shows discrimination of the samples regarding the species and hydric stress (*P. amarus* samples are shown in blue and *P. niruri* are in red). The numbers denote the extent of hydric stress: 0 is the non-stressed plant, 3 is three days of hydric stress, 5 is five days of hydric stress, 7 is seven days of hydric stress, and 10 is ten days of hydric stress. Figure 3b shows the relevant loading projection of PC1.

The PC1 axis with 55.1% of the total variance shows that plants under low (or no) hydric stress are located at positive scores of PC1, while water-stressed plants are located at negative scores of PC1 (for both *Phyllanthus* species). As observed in the loadings graphs, the highest amounts of sucrose were found in the least stressed plant. On the other hand, the PCA presented the opposite behavior for glucose and fructose, which were found in higher concentration in water-stressed leaves, indicating the conversion of sucrose into less complex sugars such as glucose and fructose. Additionally, loadings graphs indicate higher amounts of gamma-aminobutyric (GABA), succinic, citric and malic acids, and amino acids alanine and valine in samples at negative values of PC1 (drought hydric stressed). However, the behavior of those metabolites was extensively investigated with the quantification through NMR (item 3.2.1), because the PCA only presents variation tendencies.

An in-depth evaluation was also performed for aliphatic (*δ* 0.50–3.17) and aromatic (*δ* 6.20–9.20) regions separately. Based on the aliphatic evaluation, the same clustering tendencies were observed in the entire spectra analyses (for more details, see Fig. 2 in Supplementary Information file). The PC1 and PC2 axes (88.4% of the total variance) also showed a tendency of distinction of the samples regarding the species and the hydric stress imposed. The drought stress *P. amarus* is located at positive scores of PC2 and the drought *P. niruri* is located at negative scores of PC2. Therefore, these axes show different ways that each species responds to the hydric stress condition. As can be observed in the loadings graph (Fig. 3b), *P. niruri* presented higher amount of *α-* and *β*-glucose, and *P. amarus* the highest amount of sucrose and proline. The detailed evaluation of the aromatic region (PCA graph available in Supplementary Information, Fig. 3) also presented the samples’ discrimination regarding species and hydric stress. The species under lowest (or no) hydric stress are located at negative scores of PC1, while plants subjected to drought stress are located at the positive scores of PC1. According to the loadings plots, the positive scores of PC1 are related to a decrease in the amount of aromatic and phenolic compounds, such as tyrosine, corilagin, gallic, and ellagic acids.

### ^1^H qNMR results

**Figure 4:**
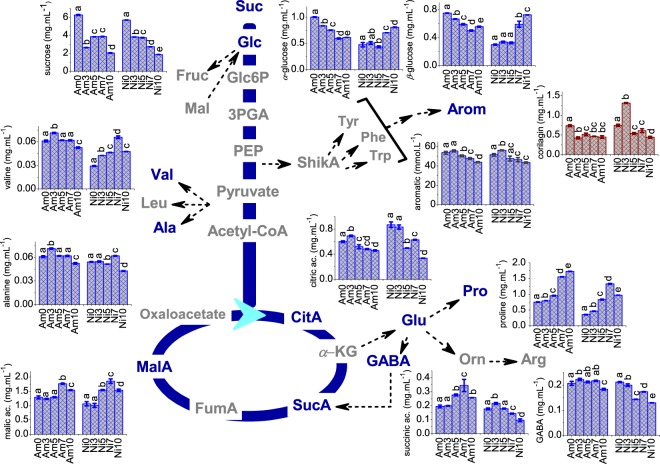
Plant metabolic pathways with the stress-related compounds quantified by ^1^H *q*NMR. Legend: Number 0 corresponds to the non-stressed plant, 3 corresponds to three days of hydric stress, 5 corresponds to five days of hydric stress, 7 corresponds to seven days of hydric stress, and 10 corresponds to ten days of hydric stress. The upper letter coefficients indicate different values at 95% of the significance level.

In order to explore the dehydration-inducible metabolome^21^, the compounds that presented high variations in the chemometrics analysis and exhibited low or non-overlapped resonances were quantified and correlated with the plant metabolic pathways (Fig. 4). When necessary, the deconvolution process was performed in order to remove problems faced with overlapped signals^22^. In addition, an estimation of the variation of the total hydrogenated aromatic organic matter during the hydric stress was performed (see detailed procedure in experimental section, item 2.3.1), which is also presented in Fig. 4. One-way ANOVA was applied to statistically certify the differences between the values, which are shown as the letter coefficients (different letters indicate different values at 95% of the significance level). Therefore, Fig. 4 gives an insight into the regulation of metabolic networks during dehydration stress.

For *P. amarus*, an accumulation of malic and succinic acids was observed until seven days of stress followed by a drop; the citric acid level initially increased (over three days of drought) and then decreased. For *P. niruri*, an accumulation of malic acid was observed after seven days of drought (*Ni7*) followed by a decrease in the maximal concentration of succinic acid on the third day of stress; citric acid (which was more concentrated in *P. niruri*) decreased after the entire period of drought. Amino acids showed a diverse trend in their concentration, depending on the species. For *P. amarus*, an accumulation of valine and alanine in *Am3* (three days of hydric stress) was detected, followed by a decline over the remaining days. For GABA, a major decline was noted with 10 days of hydric stress (*Am10*), and for proline, a constant increase was observed, reaching the maximal concentration at ten days. For *P. niruri*, alanine and valine content increased after 7 days of drought (*Ni7*), followed by a decrease at 10 days (*Ni10*). For GABA, a significant decline was observed at five days of hydric stress (*Ni5*); accumulation of proline occurred until seven days of drought (*Ni7*) followed by a decrease, which indicates that after ten days of drought, the plant was recovering from the stress^3^. For carbohydrate content in *P. amarus*, the sucrose content declined with three days of drought (*Am3*), slightly increased over five and seven days of drought (*Am5* and *Am7*), and declined again with ten days of stress (*Am10*). The glucose content decreased over ten days of drought (*Am10*). The sucrose content in *P. niruri* continually decreased, while the amount of glucose increased over ten days (*Ni10*). The aromatic content presented as the total hydrogenated aromatic organic matter showed the same tendencies for both *Phyllanthus* species: a slight increase with three days of drought (*Am3* and *Ni3*), followed by a decrease over the drought period.

### Chemometric evaluation of the UPLC-HRMS data

**Figure 5:**
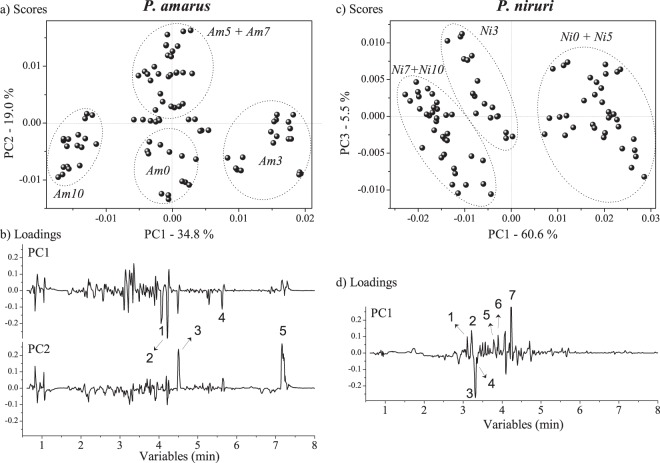
PCA results from *P. amarus* and *P. niruri* under different periods of hydric stress. (**a**,**c**) Scores plots. Legend: *Am0* and *Ni0* are non-stressed plants; *Am3* and *Ni3* correspond to three days of hydric stress, *Am5* and *Ni5* are five days of hydric stress, *Am7* and *Ni7* are seven days of hydric stress, and *Am10* and *Ni10* are ten days of hydric stress. (**b**) *P. amarus* loadings from respective axes plotted in lines form. Legend: 1 - ellagic acid; 2 - quercentin-3-*O*-hexoside; 3 - narirutin; 4 - tri-*O*-methylellagic acid; 5 - niruriflavone. (**d**) *P. niruri* loadings from respective axis plotted in lines form. Legend: 1 - brevifolin carboxylic acid; 2 - repandusinic acid A (isomer); 3 - geraniin; 4 - corilagin; 5 - orientin-2″-*O*-rhamnoside; 6 - geraniin (isomer); 7 - quercentin-3-*O*-hexoside.

In order to understand the effect of hydric stress in secondary metabolites of both *Phyllanthus* species (*P. amarus* and *P. niruri*), unsupervised multivariate analysis was employed to investigate the *UPLC-HRMS data*. Figure 5 presents the PCA results for *P. amarus* (left side) and *P. niruri* (right side): (a) and (c) are the plots of the scores, and (b) and (d) are the loadings plots with the most relevant compounds, respectively. Axes values refer to the explained variance on each principal component. Tables to summarize the MS dataset for identification of the compounds in both *Phyllanthus* species are available in Supplementary Information (Tables 2 and 3), and the most significant compounds are annotated in Fig. 5(b) and (d) as numbers.

Important information regarding the effect of drought on the secondary metabolite of *P. amarus* is presented by PC1 and PC2 axes. PC1 (34.8% of total variance at Fig. 5a) mainly discriminates the harsher water privation (10 days i.e. *Am10*) from the other samples. According to the loadings, the *Am10* samples present higher concentration of ellagic acid, tri-*O*-methylellagic acid, and narirutin. The PC2 axis (19.0% of total variance, Fig. 5a) shows positive scores for plants under five and seven days of hydric stresses (*Am5* and *Am7*) and negative scores for the other samples (*Am0*, *Am3*, and *Am10*). According to the loadings, samples under five and seven days of hydric stresses present the highest amounts of narirutin (flavanones) and niruriflavone (flavone sulfonic acid).

The PCA scores plot of *P. niruri*, PC1 (60.6% of total variance, Fig. 5a) is the most important axis for discrimination of the samples. Three clusters can be observed: in positive region of PC1 are samples under intermediary hydric stresses (*Ni0* and *Ni5*, i.e., non-stressed sample and sample submitted to five days of drought), and at null values of PC1 are samples under three days of drought (*Ni3*). Plants under the harshest hydric stresses (*Ni7* and *Ni10*, i.e., the samples submitted to seven and ten days of drought, respectively) are in the negative region of PC1. The loadings graphs show that the samples with negative scores of PC1 (*Ni7* and *Ni10*) present mainly the highest amounts of geraniin and corilagin.

**Figure 6:**
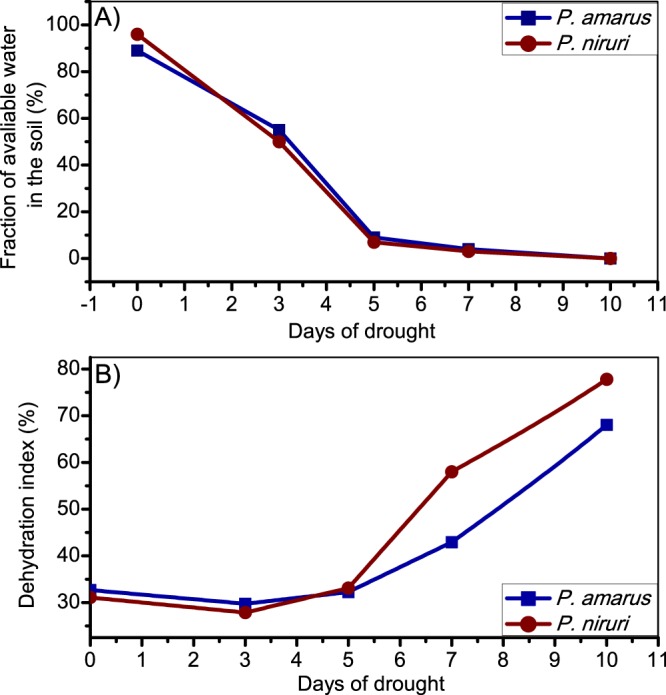
Fraction of water available in the soil during the drought period.

These results can be supported by agronomic parameters. Figure 6A shows that the fraction of water available in the soil during the drought period was similar for both species. Figure 6B presents the *P. amarus* species with higher capacity to maintain water after 5 days of drought than *P. niruri*.

A multivariate calibration was also performed for the entire NMR spectrum with the agronomic parameters of tissue and soil humidity. Table 1 presents the statistical parameters for classification and validation. Among the developed models, a better performance was observed for tissue humidity from *P. amarus*, since the SEC to SEV ratio was above 0.75, and *r*^2^ for calibration and validation (0.95 and 0.93, respectively) indicated a well-adjusted model using less latent variables (LV)^23^.

**Table 1:** PLS of NMR data versus tissue and soil humidity.

Model	n. of LV	r^2^ Cal^a^	RMSEC^b^	r^2^ Val^c^	RMSECV^d^	SEC/SEV^e^
*P. amarus*						
*Tissue humidity*	2	0.95	3.11	0.93	4.06	0.77
*Soil humidity*	2	0.92	2.10	0.85	3.20	0.65
*P. niruri*						
*Tissue humidity*	3	0.97	3.09	0.93	5.28	0.58
*Soil humidity*	3	0.96	1.51	0.92	2.29	0.66

^a^Coefficient of correlation between the real concentration and the concentration predicted during the calibration; ^b^Root Mean Square Error of Calibration; ^c^Coefficient of correlation between the real concentration and the concentration predicted during the validation; ^d^Root Mean Square Error of Cross Validation; ^e^Similarity criterion.

## Discussion

Hydrogen peroxide (H_2_O_2_) is a reactive oxygen species (ROS) that, under oxidative stress conditions as imposed by drought, acts as a signal inducing the antioxidant response mechanisms, as observed in the PCA of the oxidative/antioxidant metabolism of the drought stressed species (item 3.1, Fig. 1). The H_2_O_2_ (PER) is correlated to the biological membrane lipid peroxidation (PER_LIP) degree (parameter of membrane integrity) leading to tissue disintegration. However, in response to such oxidative imbalance, cells produce antioxidants of different chemical natures to eliminate or neutralize reactive species as antioxidant enzymes. The antioxidant enzyme superoxide dismutase (SOD) scavenges radical superoxide (O_2_^−^) converting into H_2_O_2_, which is, subsequently, neutralized by catalase (CAT) or ascorbate peroxidase (APX) to water and oxygen. The higher levels of PER, PER_LIP, SOD and APX activities found in *P. amarus* corroborate the hypothesis that an enzymatic antioxidant system is induced by an oxidative imbalance. Moreover, the endogenously higher levels of *P. amarus* represent an adaptation of the plant to drought that reflects the small behavioral difference observed among control (*Am0*) and stressed plants (*Am3* and *Am10*) (Fig. 1), which possibly confers tolerance. Meanwhile, *P. niruri* exhibits alterations in metabolism or homeostasis even at lower levels of water stress (*Ni3* and *Ni5*), an indicator of a greater sensitivity, thus engaging in acclimation to such stressful conditions.

The HCA of the ^1^H NMR data indicates that the imposed stress conditions are inducing metabolic adjustments in response to the adverse environmental circumstances. In order to observe the more clearly the metabolites within the stress conditions, the PCA was performed. In summary, a hydric stress based change in the content of several compounds, such as carbohydrates, amino acids, organic acids, and phenolic compounds, was observed.

The quantitative analysis by ^1^H NMR (item 3.3, Fig. 4) was important for observing the stress-related metabolites acting as indicators of stress extension; they played an important role in drought response through a range of complex regulatory events and allowed the plant to recognize and transduce external signals. The ANOVA evaluation of the ^1^H *q*NMR results indicated that the variables identified by PCA are statistically significant for the distinction of the samples. It is known that water stress directly affects the water transport system across membranes and the photosynthetic process, is mediated by stomatal closure^8,24^. Therefore, plant metabolisms recognized and transduced the adverse external environmental conditions in order to trigger adaptive responses^25–27^. Overall, the net content of the quantified organic acids was higher in *P. amarus* than in *P. niruri*. Predominantly, the changes observed in the levels of malic, citric, and succinic organic acids differed based on the species and the hydric stress level. Organic acids are intermediates involved in the tricarboxylic acid (TCA) cycle for energy production^28^, assimilation of carbon and nitrogen, and osmotic regulation^29^; recent studies have correlated the accumulation of organic acids with improvement of drought tolerance^30–32^. According to the amino acids, *P. amarus* presented a higher content of valine and leucine, a reduced decline in the GABA content, and the most pronounced increase in the proline content (and also further increase in concentration under normal growth). The accumulation of amino acids improves stress tolerance in plants^33,34^. Fait *et al*.^35^ shows a close relationship between the deficiency of GABA and the accumulation of reactive oxygen intermediates. In addition, proline improves osmotic regulation, induces antioxidant protective pathways^36^, and accumulates as a drought response; for this reason, it has been strongly correlated with stress tolerance^3^. Therefore, the aforementioned results corroborate the idea that *P. amarus* is better adapted to drought.

Plants accumulate carbohydrates to maintain cell turgor, and sucrose protects membranes and proteins from stress injury^37,38^. However, drought decreases the photosynthetic rate and carbohydrate metabolism in leaves^39^. For *P. amarus*, sucrose and glucose contents decreased during drought, which may be correlated to an increase in the intake of the plant feedstock (as carbohydrate) to support the recovery of growth. For *P. niruri*, although the sucrose content decreased, the glucose level increased as a response to stress, which showed that this species might adjust its metabolism in order to increase the osmotic regulation. However, *P. amarus* presented a higher content of carbohydrates in non-stressed control plants, suggesting once again that this species is better adapted to drought than *P. niruri*.

Some authors have reported an increase of phenolic content of the plant under stress^40^, while others have suggested a decline over long periods of drought^6,41^. It is known that phenolic compounds protect plants against oxidation by scavenging reactive oxygen species^42^. For *Phyllanthus* (regardless of the species), a slight increase was observed over a short-term period (three days), followed by a drop of phenolic content over long-term drought. In addition, corilagin is overexpressed in *P. niruri* on the third day of drought (*Ni3*), which indicates that short periods of water deficit induce the production of this compound. Corilagin (*β*-1-*O*-galloyl-3,6-(R)-hexahydroxydiphenoyl-D-glucose) is a tannin with pharmacological properties, such as antiatherogenic^43^, hepatoprotective, and antioxidant properties^44^.

UPLC-HRMS was employed to detect minor secondary metabolites, which are not detectable with NMR, offering important complementary information. As described above, phenolic content increased upon drought in order to protect plants against oxidation^42^. Therefore, the increase in ellagic acid and tri-*O*-methylellagic acid might be correlated with a high increasing of proline (Fig. 5) in *P. amarus*, which results in an increasing of drought tolerance for this species. The action of ellagic acid was revealed in 2013 by El-Soud and co-workers, who showed that ellagic acid induced a reduction of osmotic stress on chickpea seeds by increasing antioxidant capacity, as well as increasing the amount of osmoprotectants such as proline and glycine betaine^45^. Additionally, the increase of highly modified flavonoid glicosides such as quercentin-3-*O*-hexoside (that present radical scavenging activities^46^) in highly stressed plants (*Am10*) shows up-regulation in order to protect them against oxidation. The PC2 axis (Fig. 5c) shows plants under five and seven days of hydric stresses (*Am5* and *Am7*) in the positive region. According to the loadings, samples under five and seven days of hydric stresses presented the highest amounts of narirutin (flavanones) and niruriflavone (flavone sulfonic acid), which are known as antioxidant compounds^47^. This fact indicates that in response to the drought, *P. amarus* increases its phenolic content in order to protect cellular assemblies from oxidative damage. In addition, these results are in agreement with the higher content of the catalase enzyme in these plants. Geraniin and corilagin are ellagitanins with high anti-oxidant and therapeutic activity^48,49^. The drought induced *P. niruri* to produce more complex substances such as the ellagitanins, an important class of compounds to the therapeutic activity of *P. niruri*. Hence, these results were corroborated by *q*NMR (Fig. 5), which showed that content of corilagin was higher in samples exposed to three days of drought. Both data indicated that the biosynthesis of ellagitanins might be induced under specific drought conditions, and therefore the bioactivity of the species.

For agronomic parameters a rapidly decreasing fraction of water available in the soil was observed up to five days after suspending irrigation, remaining practically stable after that. The soil water deficit directly affected the plant metabolites pathways due to plant dehydration. Therefore, until five days of drought, both *Phyllanthus* species are able to maintain similar water content within the plant (Fig. 6B). However, *P. amarus* is capable of maintaining higher levels of water content (or less dehydration indexes) at more extensive periods of water deficit than *P. niruri*. This evaluation corroborated the results showed in metabolite and physiologic analyses, which demonstrated that *P. amarus* presented a higher content of osmoprotectants (i.e. higher content of valine, leucine, less decrease in GABA content, and most pronounced increasing of proline) even in normal growth.

In general, a great variation of primary metabolites was observed in both species of *Phyllanthus* assessed through NMR. For this reason, a multivariate calibration was built with NMR data and agronomic parameters. This result corroborates the previous data which show *P. amarus* as a more adapted species to hydric stress, with a higher content of osmoprotectants and a higher adaptability pattern, since it correlates more with tissue humidity.

In conclusion, the physiological studies, as well as ^1^H *q*NMR and UPLC-HRMS combined with chemometrics and detailed metabolomics analysis, have been demonstrated to be powerful tools to study plants’ responses to drought and assess their tolerance. Drought induced several metabolic changes in the studied species; however, *P. amarus* was better adapted due to endogenous or drought-induced higher contents of osmoprotectants such as valine, alanine, GABA and proline. On the other hand, the behavior of *P. niruri* resembled an acclimation strategy, with stress-induced increases in glucose, proline, and ellagitannins as corilagin. An interesting aspect is that short-term drought stress induced health-promoting corilagin in *P. niruri* plants. Therefore, the physiological and biochemical responses of *Phyllanthus* species to drought stress are of relevance due to their health-promoting hepatoprotective properties.

## Methods

### Sample preparation

Plants of *Phyllanthus amarus* and *Phyllanthus niruri* were grown in a greenhouse at Embrapa Agroindustria Tropical (Fortaleza-CE, Brazil) and submitted to different levels of drought stress before the evaluations. The experiment was carried out with plants arranged in a completely randomized split-plot design, with the species in the whole-plots, the levels of soil water deficit in the split-plots, and six replications. Each split-plot consisted of four plants cultivated in pots with volume of 1.8 L, which resulted in 24 biological replicates for each treatment. The pots contained a mixture of soil and humus in a ratio 3:1, which had a field capacity of 0.332 dm^3^·dm^−3^ and a permanent wilting point of 0.022 dm^3^·dm^−3^. *Phyllanthus* seedlings were grown in trays containing the plant substrate and transplanted at 42 days after sowing. The pots were drip irrigated daily, refilling soil water content to field capacity. Irrigation was suspended 35 days after the transplanting, when all plants were at the flowering stage. Leaves and stems of the four plants of each split-plot were cut at 0, 3, 5, 7, and 10 days after irrigation was suspended. Plant materials were then weighed on an electronic scale, dried in an air forced oven at constant temperature of 40 °C for 72 h, weighed again for determination of tissue humidity, and stored. The soil water content of each pot at harvest was determined gravimetrically by drying the soil samples in an oven at constant temperature of 105 °C for 24 h.

### Physiological variables: oxidative stress and constituents of antioxidant metabolism

Assays of hydrogen peroxide (H_2_O_2_) levels, an oxidative stress marker, were determined based on previous study^50^. Tissue (0.5 g) was homogenized in an ice bath with 5 mL of trichloroacetic acid (TCA) 5% (w/v) and centrifuged at 12,000 *g* for 15 min at 4 °C. The 0.5 mL supernatant was added to 0.5 mL of 10 mM potassium phosphate buffer (pH 7.0), and 1 mL of 1 M of KI. Optical density was monitored at 390 nm and H_2_O_2_ content was given on a standard curve expressed as μmol.kg^−1^.

Biological membrane integrity was estimated by lipid peroxidation degree, which was determined by malondialdehyde (MDA) content based on the previously described method^51^. Tissue (2 g) was homogenized in 5 mL of TCA 0.1% (w/v) and centrifuged at 3,300 *g* for 20 min, at 4 °C. The supernatant (750 µL) was collected and added to 3 mL of thiobarbituric acid (TBA) 0.5% (w/v) and 20% TCA, and incubated at 95 °C for 30 min. Following the incubation, tubes were immediately cooled in an ice bath and centrifuged at 3,000 *g* for 10 min. The optical density was measured at 532 nm, and corrected for unspecific turbidity by subtracting from absorbance at 600 nm. The thiobarbituric acid reactive substances (TBARS) as MDA content was calculated using an extinction coefficient of 155 mmol.cm^−1^ and expressed as nmol MDA.kg^−1 ^^52^.

Activity of antioxidant enzymes was determined using 1 g of tissue as enzymatic extract, which was macerated for 5 min with ice-cold (distilled water) in a buffer solution (pH 8.0) containing 0.05 M tris-HCl and 0.1 mM ethylenediaminetetracetic acid (EDTA)^53^. Then, homogenates were centrifuged at 12,000 *g* for 15 min at 4 °C and the supernatant was used as crude enzyme extract. All the procedures were performed at 4 °C.

Superoxide dismutase (SOD, EC 1.15.1.1) activity was determined by spectrophotometry, based on inhibition of the photochemical reduction of nitroblue tetrazolium chloride (NBT)^54^. Optical density was measured at 560 nm and one unit enzyme activity (UEA) was defined as amount of enzyme required to cause 50% inhibition in the NBT photoreduction rate^55^, with results expressed as UAE.mg^−1^. P. Catalase (CAT, EC 1.11.1.6) activity was measured according to a previous study^56^. Decreases in H_2_O_2_ content were monitored by measuring optical density at 240 nm and quantified using the molar extinction coefficient (36 M.cm^−1^), with results expressed in µmol H_2_O_2_.min^−1^.mg^−1^. P. Ascorbate peroxidase (APX, EC 1.11.1.1) activity was assayed according to a previous study^57^. The reaction was started by adding ascorbic acid, and ascorbate oxidation was measured by optical density at 290 nm, using the molar extinction coefficient for ascorbate (2.8 mM.cm^−1^), considering that 1 mol of ascorbate is required for reduction of 1 mol H_2_O_2_, with results expressed as µmol H_2_O_2_.min^−1^.mg^−1^ P.

#### Chemometric analysis of oxidative stress and constituents of antioxidant metabolism

This procedure aimed to correlate the drought conditions with the discrete variables ascorbate peroxidase (APX); hydrogen peroxide (PER); lipid peroxidation degree (PER_LIP); superoxide dismutase (SOD); catalase (CAT). Therefore, principal component analysis (PCA) was performed using the Single Value Decomposition (SVD) algorithm to decompose the matrix, and autoscaling was applied to provide the same importance to the variables (average zero and variance equal to one).

### NMR spectroscopy and molecular identification

The 24 biological samples (of each group) were divided into three groups (of 8 replicates each) for NMR analysis. From those groups, 30 mg of powdered leaves from each hydric stress level were suspended in 600 μL of D_2_O/sodium-3-trimethylsilylpropionate (TMSP-d_4_) from a stock solution (0.17 mg·mL^−1^), sonicated for 1 min and centrifuged (5 min at 605 *g*). The supernatants were transferred to 5 mm NMR tubes. The NMR experiments were performed on Agilent 600-MHz spectrometer equipped with a 5 mm (H-F/^15^N-^31^P) inverse detection One Probe™ with actively shielded Z-gradient. The ^1^H NMR spectra were acquired using PRESAT pulse sequence for water suppression (*δ* 4.79) after saturation profiling of non-deuterated water^58^. The data were acquired in triplicate with calibrated pulses, 32 scans, 64k of time domain points with a spectral window of 16.0 ppm, acquisition time of 5.0 s, and a relaxation delay of 20.0 s. The temperature was controlled at 298 K and TMSP-d_4_ was used as an internal standard (*δ* 0.0). The spectra were processed by applying exponential multiplication of the FIDs by a factor of 0.3 Hz and Fourier transformation of 32k points. Phase correction was manually performed and the baseline correction was applied over the entire spectral range.

The identification of the constituents from the *P. amarus* and *P. niruri* leaves was performed using the ^1^H-^1^H *g*COSY, ^1^H-^13^C *g*HSQC, and ^1^H-^13^C *g*HMBC experiments (molecular structures, ^1^H and ^13^C chemical shifts, multiplicity, constant coupling, 2D-NMR data acquisition and processing are available in Supplementary Information). The results were compared with existing data in open access databases and literature reports (see Supplementary Information).

#### Chemometric and quantitative analysis of the NMR data

The ^1^H NMR spectra were converted to American Standard Code for Information Interchange (ASCII) files for construction of the numerical matrices, which were used as input data to The Unscrambler^™^ 10.4 program to perform unsupervised chemometric methods by Hierarchical Cluster Analysis (HCA) and the Principal Component Analysis (PCA), in order to reduce the dimensionality of the original data and to create an overview showing trends, groupings and outliers with a confidence level of 95%^59^. For HCA, the Ward’s method using Squared Euclidean distance was applied. PCA analyses were performed using the Single Value Decomposition (SVD) algorithm to decompose the matrix, and three groups of ^1^H NMR spectra were evaluated: total spectra (*δ* 0.50 to 9.20); aliphatic region (*δ* 0.50 to 3.17); and aromatic region (*δ* 6.20 to 9.20). The area influenced by non-deuterated water suppression (*δ* 4.85 to 5.15 – according to the saturation profile analysis) was excluded. Afterward, misalignments of the spectra were adjusted by a Correlation Optimized Warping (COW) algorithm using a segment of 20 data points and a slack of 10 data points^60^. Baseline correction using a linear fit algorithm and normalization processing were applied over the spectra to reduce analytical errors and spectrum noise. Mean-centered processing was applied over the variables (composition) to provide better differences among samples^61,62^.

The metabolites valine (characteristic ^1^H chemical shift at *δ* 1.07), alanine (*δ* 1.50), proline (*δ* 2.11), succinic acid (*δ* 2.50), citric acid (*δ* 2.67), GABA (*δ* 3.03), malic acid (*δ* 4.37), *α* and *β*-glucose (*δ* 5.21 and *δ* 4.64, respectively), sucrose (*δ* 5.42), corilagin (*δ* 7.06) and also the whole aromatic region (*δ* 6.20 to 9.20) were quantified by the external reference method. In this method, a known amount of a standard solution of sucrose was used to calibrate the equipment. Consequently, the probe file was updated with all the parameters required for concentration determination of an unknown sample.

The estimation of the variation of total aromatic organic matter (between *δ* 6.20 ppm and *δ* 9.20) aimed to obtain a variation profile of total hydrogenated organic compounds such as alkenes, heteroaromatic, and aldehydic protons. Consequently, all of the signals were integrated and the reduction values are the association of each sample under hydric stress with the control sample for both *Phyllanthus* species.

A multivariate PLS calibration modeling was also developed to evaluate the classification and prediction capabilities of the NMR data (total NMR spectra) with the agronomic parameters of tissue and soil humidity, with a confidence level of 95%. The NIPALS (nonlinear iterative partial least squares) algorithm was applied to construct the PLS models and the statistical parameters, as the figures of merit were shown to be RMSEC (root mean square error of calibration); RMSEV (root mean square error of validation); coefficient of correlation for calibration (r^2^ cal) and validation (r^2^ val); and similarity criterion SEC/SEV^61^. The same preprocessing applied for PCA was applied to PLS modeling using the full leave-one-out cross-validation for both matrices.

#### Statistical evaluation of the qNMR results

The combined uncertainty of the method and the differences among the concentrations were estimated and statistically certify based on the analytical errors of the method and the standard deviation from the triplicate analyses. The data obtained were subjected to one-way analysis of variance ANOVA using Origin^™^ 9.4 software, with significance level of 0.05, means comparison using Tukey’s test, and Levene’s to test the homogeneity of variance, in order to statistically certify the differences in the variation values.

### Sample preparation and UPLC-HRMS analysis

The methodology used was adapted from previous report^63^. For this experiment, the four plants cultivated in different pots were assembled given six biological replicates. From each replicate, 50 mg of ground material was extracted using 4 mL of hexane PA, being homogenized in vortex for 1 min and taken to the ultrasonic bath for 20 min with a fixed power of 135 W. The extracts were further partitioned using 4 mL of ethanol/water (70:30), homogenized in vortex for 1 min and taken to the ultrasonic bath for 20 min with a fixed power of 135 W in order to extract the polar compounds. The tubes were centrifuged for 10 min at 1008 g. Finally, a 1 mL aliquot of the lower phase (polar) was withdrawn and filtered (0.20 μm PTFE). The filtrate was collected in vials and stored (at −80 °C) for further UPLC analysis.

UPLC-HRMS analysis was performed on an Acquity system (Waters) coupled with quadrupole/TOF (Waters) equipped with an ESI source operated in the positive ion mode. The chromatographic separation was performed using a Waters Acquity UPLC BEH (150.0 × 2.1 mm, 1.7 μm) column with temperature set at 40 °C. Water and acetonitrile were used for the mobile phase, both with 0.1% of formic acid. The gradient ranged from 2 to 95% of water in 15 min in a flow of 0.4 mL·min^−1^ and injection volume of 5.0 μL per sample. N_2_ was used as desolvation gas. The desolvation temperature was set at 350 °C with flow rate of 350 L·h^−1^ and source temperature of 120 °C. The capillary voltage was set to 3,200 V. The collision energies/cone voltages were set at 6 eV/15 V (low) and 30–50 eV/30 V (high) to achieve sufficient fragmentation. Data were collected in triplicate using negative and positive ionization modes between 100 and 1180 Da, and the mode tandem was MS^E^.

#### Chemometric analysis of the UPLC-HRMS data

Chemometric analysis was performed to understand the secondary metabolic variation induced by different periods of hydric stress. The analyses were performed using the region between zero and 8 min of the chromatogram resulting in a matrix with dimensionality of 74,070 data points for each *Phyllanthus* species (90 samples × 823 variables). The same statistical parameters and processing mentioned at Section 2.3.1 were applied to the samples and variables to test the robustness and accuracy of the models^61^.

## Electronic supplementary material

Supplementary Information

## Data Availability

The datasets generated during and/or analyzed during the current study are available from the corresponding author on reasonable request.
