# Supplementary material for: Physiological changes for drought resistance in different species of Phyllanthus
Source: Sci Rep. 2018 Oct 11;8:15141. doi: 10.1038/s41598-018-33496-7 (PMC6181946; doi:10.1038/s41598-018-33496-7)
Supplement: Supplementary file 1 — Supplementary Information [file 41598_2018_33496_MOESM1_ESM.pdf]

Physiological changes for drought resistance in different species of *Phyllanthus*

Elenilson G. Alves Filho[a]; Luiza N. Braga[b]; Lorena Mara A. Silva[a]; Fábio R. Miranda[a]; Ebenézer O. Silva[a]; Kirley M. Canuto[a]; Maria Raquel Miranda[c]; Edy S. de Brito[a]; Guilherme J. Zocolo[a]

[a] Embrapa Agroindustria Tropical, Fortaleza-CE, Brazil.
[b] Departamento de Agronomia, Universidade Federal do Ceará, Fortaleza-CE, Brazil.
[c] Departamento de Bioquímica e Biologia Molecular, Universidade Federal do Ceará, Fortaleza-CE, Brazil.

* Corresponding author:
E-mail: guilherme.zocolo@embrapa.br
Phone: +55 (85) 3391-7360

1. NMR data from the identification of the organic compounds in *P. amarus* and *P niruri* species

Table 1 presents the structures, ^{1}H and ^{13}C chemical shifts (*δ*), multiplicity, correlations, and constant coupling (*J* in HZ) of the primary metabolites identified in both *Phyllanthus* species, with characteristics signals at: aliphatic region (between *δ* 0.66 to 2.60) from aliphatic alicyclic, allylic, *β*-substituted aliphatic, and alkyne protons; carbinolic region (between *δ* 2.60 to 5.50) from olefinic, *α*-monosubstituted and *α*-disubstituted aliphatic protons; and aromatic region (between *δ* 6.80 to 9.22) from alkene, heteroaromatic and aldehydic protons [1-3]. Two-dimensional (2D) NMR experiments were acquired using the standard spectrometer library pulse sequences. The ^{1}H-^{1}H *g*COSY experiments were obtained with spectral width of 18,028.1 Hz in both dimensions; 1442 × 200 data matrix; 32 scans per t1 increment and relaxation delay of 1.0 s. The one-bond ^{1}H-^{13}C *g*HSQC experiments were acquired with an evolution delay of 1.7 ms for an average ^{1}J(C,H) of 145 Hz; 1442 200 data matrix; 80 scans per t1 increment; spectral widths of 9615.4 Hz in f2 and 30,165.9 Hz in f1, and relaxation delay of 1.0 s. The ^{1}H-^{13}C *g*HMBC experiments were recorded with an evolution delay of 50.0 ms for ^{LR}J(C,H) of 10 Hz; 1442 × 200 data matrix; 180 scans per t1 increment; spectral widths of 9615.4 Hz in f2 and 30,165.9 Hz in f1, and relaxation delay of 1.0 s.

Table 1 – Primary metabolites identified in *P. Amarus* and *P. niruri*.

Structures	*δ ^{1}H (multip.* J in Hz)*	*δ ^{13}C*	*δ ^{1}H ref.*	*δ ^{13}C ref.*
AMINO ACIDS				
Alanine 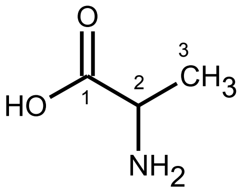	2 - 3.81 (o)<br>3 - 1.50 (*d* 7.2)	53.5<br>19.0	3.90 (*q* 7.3)<br>1.52 (*d* 7.3)	53.4<br>19.1
GABA (gamma-aminobutyric) 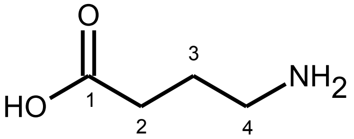	4 - 3.03 (*m*)<br>3 - 1.93 (*m*)<br>2 - 2.33 (o)	42.2<br>26.4<br>37.0	2.99 (*t* 7.7)<br>1.88 (*qui* 7.6)<br>2.28 (*t* 7.3)	42.2<br>26.3<br>37.1
Glycine	2 - 3.81 (o)	46.4	3.54 (*s*)	44.3

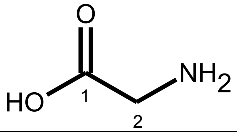				
Lysine 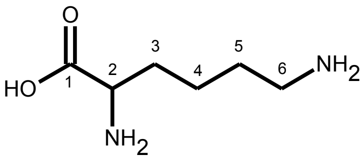	4 - 1.43 (*m*)<br>5 - 1.69 (*m*)<br>3 - 1.81 (*m*)<br>6 - 2.86 (o)<br>2 - 3.56 (o)		1.49 (*m*)<br>1.72 (*m*)<br>1.88 (*m*)<br>3.02 (*t*)<br>3.75 (*t*)	24.0<br>29.1<br>32.6<br>42.1<br>57.4
Serine 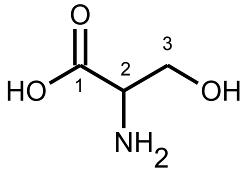	2 - 3.80 (o)<br>3 - 3.87 (o)	57.1<br>58.9	3.83 (*dd* 5.6, 3.8)<br>3.95 (*m*)	59.2<br>63.1
Tyrosine 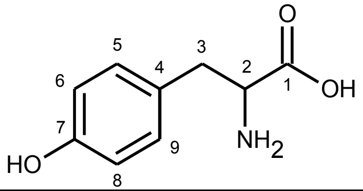	6,8 - 6.89 (*d*)<br>5,9 - 7.18 (o)<br>3 - o<br>2 - o	119.1<br>133.1<br>o<br>o	6.84 (*d* 8.5)<br>7.17 (*d* 8.5)<br>3.17 (n)<br>3.93 (n)	118.9<br>133.5<br>38.3<br>59.0
Valine 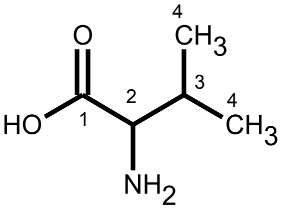	2 - 3.62 (o)<br>3 - 2.36 (o)<br>4 - 1.01 (*d* 7.2)<br>5 - 1.06 (*d* 7.2)	o<br>32.0<br>19.6<br>21.0	3.82 (*d* 4.4)<br>2.33 (*m*)<br>1.02 (*d* 7.1)<br>1.06 (*d*7.1)	n<br>32.0<br>19.1<br>20.9

ORGANIC ACIDS

Citric 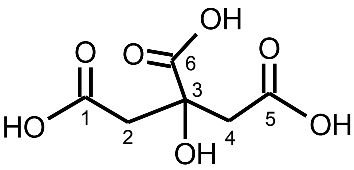	2 - 2.43 (o)<br>4 - 2.70 (o)	45.4<br>45.4	2.52 (*d* 15.8)<br>3.66 (*d* 15.8)	48.6<br>48.6
Ellagic 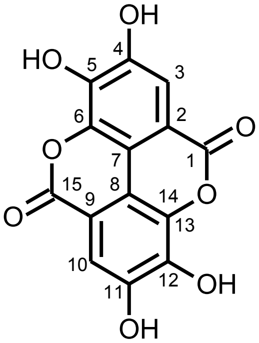	3,10 - 7.48 (*s*)<br>2 - no<br>4 - no<br>5 - no	114.6<br>114.8<br>147.2<br>157.9	7.58 (*s*)<br>no<br>no<br>no	113.5<br>115.7<br>146.7<br>159.1

Gallic<br>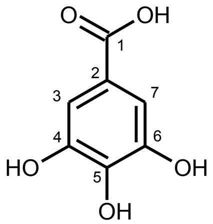	3,7 - 7.00 (*s*)<br>2 - no<br>4,6 - no<br>5 - no<br>1 - no	113.6<br>118.2<br>140.0<br>147.4<br>171.4	7.04 (*s*)<br>no<br>no<br>no<br>no	112.2<br>123.9<br>138.2<br>147.4<br>167.4
Malic<br>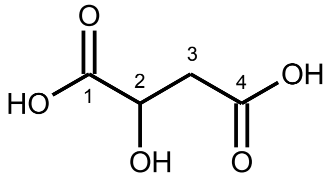	3 - 2.51 (*m*)<br>3 - 2.77 (*m*)<br>2 - 4.33 (*m*)	48.5<br>48.5<br>74.9	2.68 (*dd*)<br>2.85 (*dd*)<br>4.28 (*m*)	45.5<br>45.5<br>73.2
Mucic<br>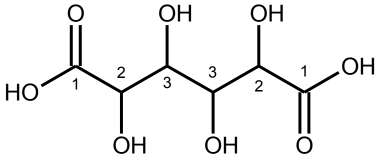	1 - no<br>2 - 4.01 (o)<br>3 - 4.31 (o)	182.7<br>74.2<br>74.4	 3.94 (*s*)<br>4.25 (*s*)	180.3<br>74.5<br>74.2

CARBOHYDRATES

Fructose<br>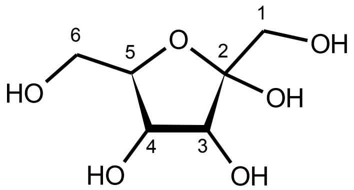	3 -<br>4 -<br>5 -<br>6' -<br>6" -<br>1 -		4.10 (*m*)<br>4.10 (*m*)<br>3.82 (*m*)<br>3.80 (*m*)<br>3.67 (*m*)<br>3.59 (*m*)	78.2<br>77.4<br>83.6<br>65.4<br>65.6<br>65.6
α-glucose<br>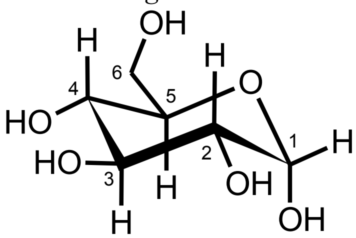	1 - 5.25 (*d* 3.8)<br>2 - 3.48 (o)<br>3 - 3.79 (o)<br>4 - 3.52 (o)<br>5 - 3.70 (o)<br>6 - 3.88 (o)	95.0<br>72.5<br>75.1<br>74.3<br>64.4<br>74.5	5.25 (*d* 3.8)<br>3.89-3.36 (o)<br>n<br>n<br>n<br>n	95.4<br>72.2<br>76.0<br>72.8<br>64.2<br>74.5
β-glucose<br>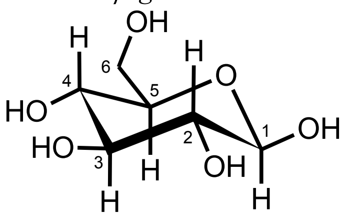	1 - 4.67 (*d* 7.90)<br>2 - 3.27 (*m*)<br>3 - 3.71 (*m*)<br>4 - 3.50 (*m*)<br>5 - 3.43 (*m*)<br>6 - 3.87 (*m*)	98.9<br>77.9<br>64.0<br>79.0<br>72.4<br>63.9	4.66 (*d* 8.1)<br>3.25 (*t* 8.4)<br>n<br>n<br>n<br>n	99.2<br>77.6<br>56.1<br>79.0<br>72.8<br>63.1
Sucrose	1 - 5.43 (*d* 3.7)<br>2 - 3.56 (o)<br>3 - 3.76 (o)<br>4 - 3.48 (o)<br>5 - 3.85 (o)<br>6 - 3.82 (o)<br>1' - 3.82 (o)	95.2<br>74.5<br>75.7<br>72.1<br>75.8<br>62.9<br>65.4	5.44 (*d* 3.8)<br>3.89-3.57 (*m*)<br>3.74 (m)<br>3.47 (m)<br>3.83 (m)<br>3.81 (m)<br>3.66 (m)	94.7<br>73.5<br>75.3<br>71.8<br>74.9<br>62.8<br>64.0

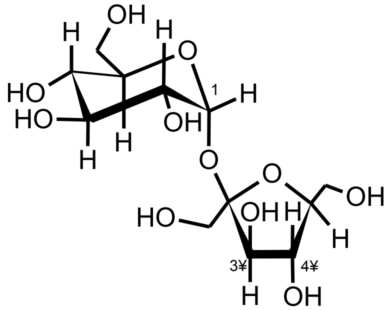	2' - no<br>3' - 4.22 (*m*)<br>4' - 4.05 (*m*)<br>5' - 3.89 (o)<br>6' - 3.68 (o)	104.7<br>79.3<br>77.0<br>84.1<br>64.8	no<br>4.20 (*t* 8.4)<br>4.08 (*d* 9.0)<br>3.87 (m)<br>3.81 (m)	106.3<br>79.0<br>76.6<br>84.0<br>65.0

OTHER COMPOUNDS

Corilagin<br>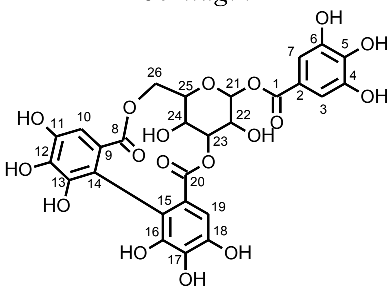	3,7 - 7.07 (*s*)<br>19 - 6.50 (o)<br>10 - 6.45 (o)	112.1<br>105.3<br>105.6	6.98 (*s*)<br>6.53 (*s*)<br>6.43 (*s*)<br>6.17 (*d* 7.5)<br>4.58 (*sl*)<br>4.36 (*t* 8.1)<br>4.27 (*d* 3.0)<br>4.21 (*dd* 10.8; 7.7)<br>3.99 (*dd* 10.8; 10.2)<br>3.88 (*d* 7.5)	109.0<br>105.9<br>106.9<br>92.2<br>77.5<br>76.4<br>62.2<br>63.9<br>71.7

s – simplet; *d* – duplet; *t* – triplet; *q* – quadruplet; *quin* – quintet; *dd* – double duplet; o – overlapping signal; n – no information; no – not observed

2. Chemometric evaluation of the NMR data

The unsupervised chemometric modeling by Hierarchical Cluster Analysis (HCA) applied to *P. amarus* and *P. niruri* evaluation presented natural clusters into two-dimensional space in dendrogram form (Fig. 1).

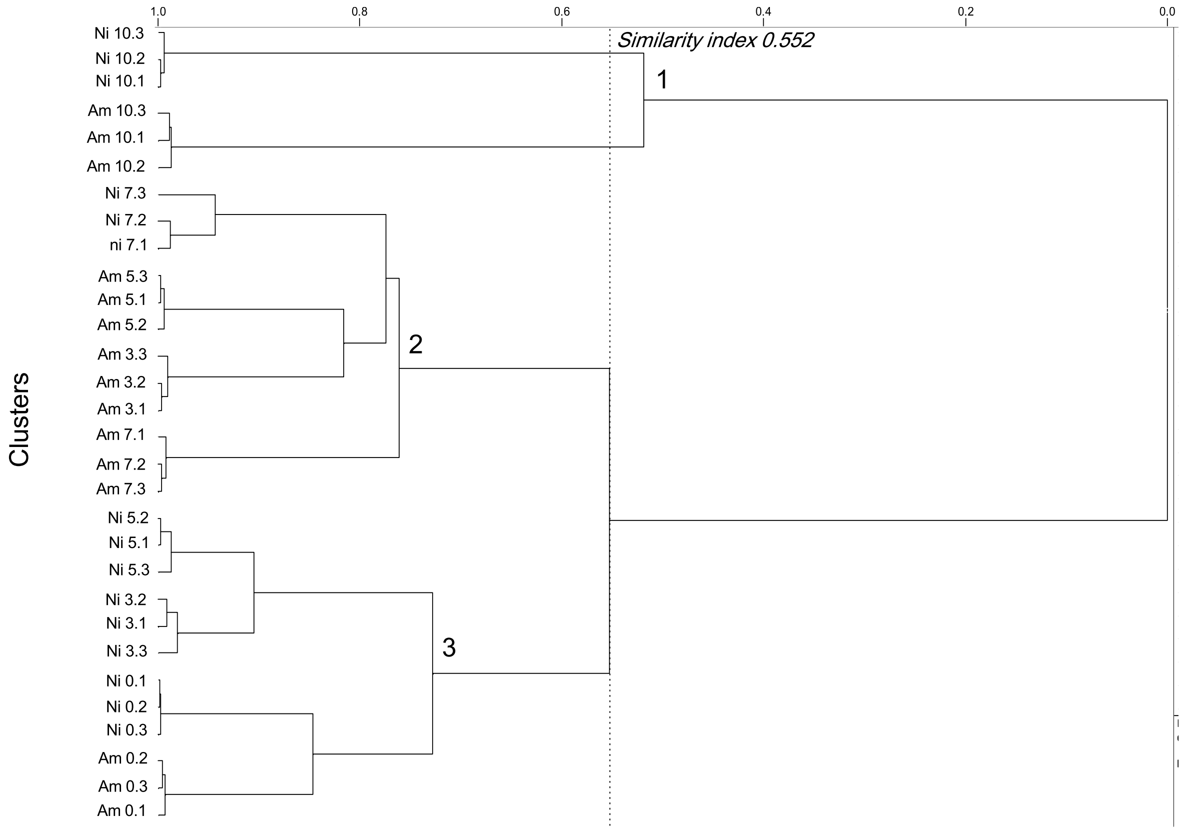

Figure 1. Dendrogram from *P. amarus* and *P. niruri* leaves analyzed by ^{1}H NMR.

Detail evaluations of the aliphatic (δ 0.50 to 3.17) and aromatic (δ 6.20 to 9.20) regions were also performed in order to get deeply information of the variability of *Phyllanthus* leaves under different hydric conditions. The results are illustrated in Fig. 2 and 3, respectively.

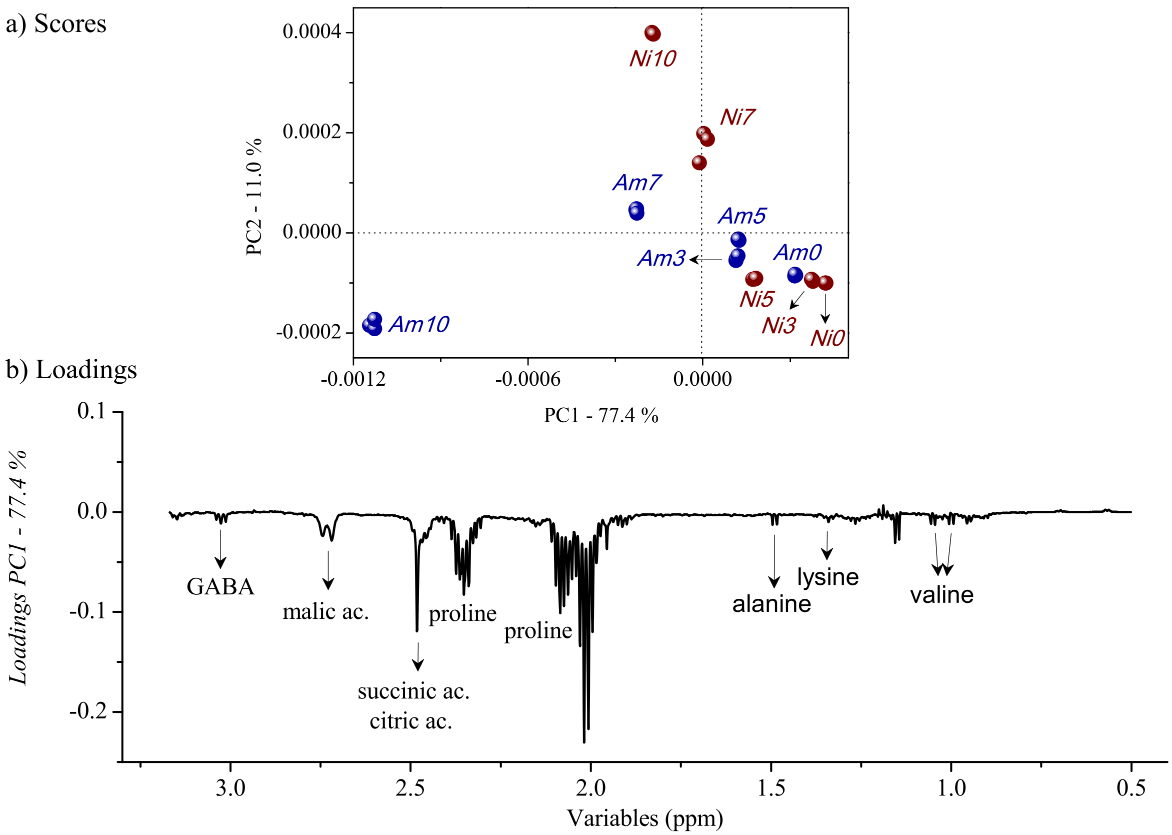

Figure 2. PCA for evaluation of aliphatic region (δ 0.50 to 3.17): a) PC1 × PC2 scores plot for *P. amarus* and *P. niruri* submitted to different hydric stress conditions, and b) PC1 loadings plotted in lines form. Legend: number 1 corresponds to the non-stressed plant, 2 corresponds to three days of hydric stress, 3 corresponds to five days of hydric stress, 4 corresponds to seven days of hydric stress, and 5 corresponds to ten days of hydric stress.

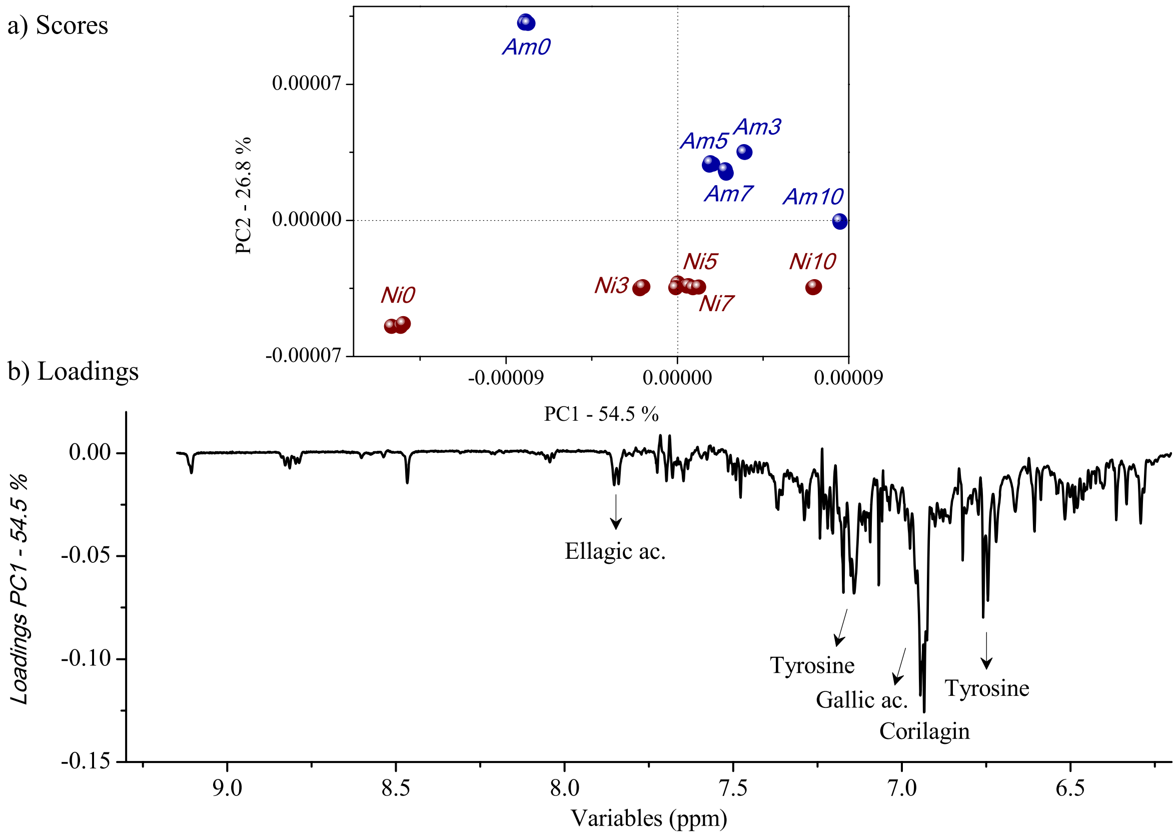

Figure 3. PCA for evaluation of aromatic region (δ 6.20 to 9.20): a) PC1 × PC2 scores plot for *P. amarus* and *P. niruri* submitted to different hydric stress conditions, and b) PC1 loadings plotted in lines form. Legend: number 1 corresponds to the non-stressed plant, 2 corresponds to three days of hydric stress, 3 corresponds to five days of hydric stress, 4 corresponds to seven days of hydric stress, and 5 corresponds to ten days of hydric stress.

3. UPLC-HRMS data from the identification of the secondary metabolites in *P. amarus* and *P niruri* species

Tables 2 and 3 summarize the MS dataset for identification of the compounds in *P. amarus* and *P. niruri* species, respectively.

Table 2. Peaks tentative assignment (with error in ppm) of the relevant secondary metabolites in *P. amarus* evaluation, with retention time (in min), molecular formula, [M-H]- ion observed and calculated, fragmentation profile (MS/MS), and identification reference.

nº	RT (min)	*m/z* [M-H]$^-$	Frag. *m/z*	Mol. form.	calc. *m/z*	error (ppm)	Compound	Ref
1	4.12	300.9952	206.0800	$C_{14}H_7O_8$	300.9984	-10.6	ellagic acid	[4]
2	4.20	463.0877	206.0806<br>300.9974	$C_{21}H_{20}O_{12}$	463.0877	0.0	quercetin-3-*O*-hexoside	[5]
3	4.51	579.1713	245.0903	$C_{27}H_{32}O_{14}$	579.1714	-0.1	narirutin	[6]
4	5.65	343.0438	328.0220	$C_{17}H_{12}O_8$	343.0454	-4.7	tri-*O*-methylellagic acid	[4]
5	7.22	363.0138	268.0353<br>347.9903	$C_{16}H_{12}O_8S$	363.0175	-10.2	niruriflavone	[7]

Table 3. Peaks tentative assignment (with error in ppm) of the relevant secondary metabolites in *P. niruri* evaluation, with retention time (in min), molecular formula, [M-H]- ion observed and calculated, fragmentation profile (MS/MS), and identification reference.

nº	RT (min)	*m/z* [M-H]$^-$	Frag. *m/z*	Mol. form.	calc. *m/z*	error (ppm)	Compound	Ref
1	3.13	291.0133	191.0364<br>219.0282<br>247.0235<br>273.0026	$C_{13}H_7O_8$	291.0141	-2.1	brevifolin carboxylic acid	[4]
2	3.23	969.0867	169.0134<br>183.0273<br>247.0228<br>300.9966<br>633.0692	$C_{41}H_{30}O_{38}$	969.0845	2.3	repandusinic acid A (isomer)	[8]
3	3.32	951.0797	169.0144<br>300.9981<br>933.0655	$C_{41}H_{28}O_{27}$	951.0740	6.0	geraniin	[4]
4	3.34	633.0711	300.9950<br>463.0508	$C_{27}H_{22}O_{18}$	633.0728	-2.7	corilagin	[4]
5	3.81	593.1506	327.0494	$C_{27}H_{30}O_{15}$	593.1506	-4.0	orientin-2´´-*O*-	[9,10]

			357.0640<br>429.0833<br>473.1087				rhamnoside	
6	3.89	951.0757	169.0134<br>300.9977<br>363.0745	$C_{41}H_{28}O_{27}$	951.0740	1.8	geraniin (isomer)	4
7	4.21	463.0877	197.0414<br>300.9975<br>316.0208	C21H20O1 2	463.0877	-7.6	quercentin-3-*O*-hexoside	5

4. References

1 Chae, H.-S. *et al.* Corilagin with inhibitory activity against NO production from Euphorbia supina. *Natural Product Sciences* **20**, 126-129 (2014).

2 Alves Filho, E. G., Silva, L. M., Teofilo, E. M., Larsen, F. H. & de Brito, E. S. 1H NMR spectra dataset and solid-state NMR data of cowpea (Vigna unguiculata). *Data in brief* **11**, 136-146 (2017).

3 Wishart, D. S. *et al.* HMDB 3.0 - the human metabolome database in 2013. *Nucleic acids research* **41**, D801-D807 (2012).

4 Kumar, S. *et al.* Rapid qualitative and quantitative analysis of bioactive compounds from Phyllanthus amarus using LC/MS/MS techniques. *Industrial crops and products* **69**, 143-152 (2015).

5 Hossain, M. B., Rai, D. K., Brunton, N. P., Martin-Diana, A. B. & Barry-Ryan, C. Characterization of phenolic composition in Lamiaceae spices by LC-ESI-MS/MS. *Journal of agricultural and food chemistry* **58**, 10576-10581 (2010).

6 Spínola, V., Pinto, J. & Castilho, P. C. Identification and quantification of phenolic compounds of selected fruits from Madeira Island by HPLC-DAD–ESI-MSn and screening for their antioxidant activity. *Food chemistry* **173**, 14-30 (2015).

7 Guo, J. *et al.* Comparison of two exploratory data analysis methods for classification of Phyllanthus chemical fingerprint: unsupervised vs. supervised

pattern recognition technologies. *Analytical and bioanalytical chemistry* **407**, 1389-1401 (2015).

8 Ogata, T. *et al.* HIV-1 reverse transcriptase inhibitor from Phyllanthus niruri. *AIDS research and human retroviruses* **8**, 1937-1944 (1992).

9 Sun, Y. *et al.* Qualitative and quantitative analysis of phenolics in Tetrastigma hemsleyanum and their antioxidant and antiproliferative activities. *Journal of agricultural and food chemistry* **61**, 10507-10515 (2013).

10 da Fontoura Sprenger, R. & Cass, Q. B. Characterization of four Phyllanthus species using liquid chromatography coupled to tandem mass spectrometry. *Journal of Chromatography A* **1291**, 97-103 (2013).
